# Modulation of miRNAs by Phytochemicals in Cerebral Ischemia: A Systematic Review of In Vitro and In Vivo Studies

**DOI:** 10.1002/ptr.70062

**Published:** 2025-08-13

**Authors:** Joanna Rzemieniec, Mirko Marino, Benedetta Mercuriali, Laura Castiglioni, Paolo Gelosa, Majeda Muluhie, Cristian Del Bo’, Patrizia Riso, Luigi Sironi

**Affiliations:** ^1^ Department of Pharmaceutical Sciences Università Degli Studi di Milano Milan Italy; ^2^ Department of Food, Environmental and Nutritional Sciences (DeFENS), Division of Human Nutrition Università Degli Studi di Milano Milan Italy

**Keywords:** apoptosis, hypoxia/ischemia, inflammation, miRNA regulation, phytochemicals, stroke therapy

## Abstract

Phytochemicals gained substantial interest for their protective action in cerebral ischemia. Additionally, there is a growing body of evidence suggesting that miRs play a role in stroke therapy. However, no systematic review to date has summarized the impact of phytochemicals on miRs modulation and their role in protecting the brain against ischemia. This systematic review aims to assess the current literature on the protective action of phytochemicals in cellular and animal models of stroke, with special focus on the miRs modulation. A literature search was conducted across three databases (PubMed, Scopus, Embase), adhering to PRISMA guidelines. Studies published in English between 2014 and 2024 were included based on relevant keywords. A total of 224 articles were excluded based on their title and/or abstract as they were deemed irrelevant. Furthermore, 4 articles were excluded due to the lack of an English or full‐text version. Finally, 14 articles were included in this review. Phytochemicals reverted ischemia‐induced changes in miRs expression; that is, they upregulated miR‐556‐3p, miR‐223‐3p, miR‐1287‐5p, miR‐214, miR‐96, miR‐450b‐5p, miR‐149‐5p, miR‐375, and downregulated miR‐145‐5p, miR‐124, miR‐128‐3p, miR‐122, miR‐181b, miR‐155, miR‐134, and miR‐128‐3p. These, in turn, led to an inhibition of apoptosis, inflammation, oxidative stress, ferroptosis, pyroptosis, and activation of Wnt and AMPK signaling. Our analysis indicates that targeting miRs with phytochemicals may inhibit the ischemic injury; thus, it represents a promising tool for the development of new therapeutic strategies for stroke. Given that all of the reviewed studies utilized cellular or animal models, further human clinical trials are necessary to confirm these findings.

## Graphical abstract

Schematic representation of how phytochemicals modulate miRNA expression to confer neuroprotection in cerebral ischemia.

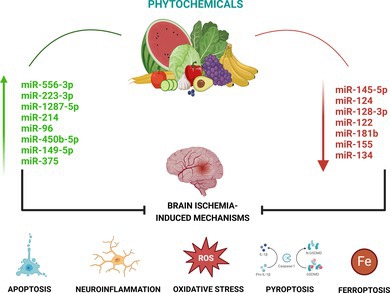

## 1 Introduction

### 1.1 Cerebral Ischemia

Stroke is the second leading cause of death worldwide, and the incidence rate of ischemic stroke will increase for both sexes and all age groups between 2020 and 2030 (Pu et al. 2023). Ischemic stroke accounts for 62.4% of all global strokes and is caused by a thrombus or embolus that obstructs the vessel supplying blood to the brain, interrupting cerebral blood flow. The brain lesions in acute ischemic stroke can be classified into two areas: ischemic core and penumbra. In the ischemic core, the cells die immediately due to energy failure that provokes necrosis. In the penumbra, the cells die mainly via apoptosis over a longer period of time (Walther et al. 2023). Therefore, the penumbra is considered the main target for pharmacological treatments. Currently, alteplase (thrombolytic drug) given within 4.5 h from symptom onset or endovascular thrombectomy performed within 24 h are the only therapies approved to treat acute ischemic stroke (Hilkens et al. 2024). However, these treatments have a long list of possible complications, including the risk of hemorrhage (Whiteley et al. 2016), angioedema (Fröhlich et al. 2019), anaphylaxis (Cheng et al. 2012) and pseudoaneurysm (Balami et al. 2018). Additionally, the treatment with alteplase has a very narrow therapeutic window (Pan and Shi 2021). Therefore, there is still a need to find safer and more efficient therapies that will protect the brain against hypoxic/ischemic damage.

In searching for new potential therapeutics and molecular targets in stroke treatment, the researchers use mainly the cellular and animal stroke models. In humans, the obstruction of the middle cerebral artery (MCA) is the most common cause of ischemia. Therefore, blocking cerebral blood flow transiently or permanently in the MCA is used to mimic ischemia in rodents (Sokolowski et al. 2023). In transient middle cerebral artery occlusion (tMCAO) the interruption of blood flow can be done by a suture inserted into the MCA or by the injection of an autologous blood clot. The occlusion has a duration typically of 60–120 min. After this time, the suture or blood clot is removed and reperfusion begins. However, in patients, transient occlusion followed by reperfusion represents only 2.5%–11.3% of all large vessel stroke patients. In the permanent MCAO (pMCAO) model, the MCA artery is occluded and no reperfusion phase appears. This happens in around 90% of cases in humans suffering from ischemic stroke (McBride and Zhang 2017). In vitro, the primary neuronal cell cultures or cell lines are subjected to oxygen and glucose deprivation (OGD) to mimic ischemia (Van Breedam and Ponsaerts 2022). This is usually conducted by exposing the cells to glucose‐free media and displacing oxygen with a nitrogen/carbon dioxide mixture in a hypoxia chamber. This model also allows for the mimicry of reperfusion conditions by reintroducing glucose with a return to atmospheric oxygen (Pilipović et al. 2023; Rzemieniec et al. 2019).

### 1.2 microRNA (miRs)

The microRNAs (miRs) discovery in 1993 in *C. elegans* significantly altered the longstanding dogmas that defined gene regulation. MiRs are very small evolutionarily conserved RNAs (~22 nucleotides) that silence gene expression by repressing translation and/or promoting mRNA degradation (Naeli et al. 2023; O'Brien et al. 2018). A recent study showed that in the brain tissue of patients suffering from stroke there is strong upregulation of miR‐1246, miR‐4516, miR‐320a‐3p, miR‐320c, miR‐204‐3p, miR‐17‐5p, miR‐16‐5p, and miR‐423‐5p; this was in agreement with the induction of the above‐mentioned miRs in stroke patient blood or CSF (Carlson et al. 2021; Li et al. 2015; Sørensen et al. 2017). The same authors also demonstrated the downregulation of miR‐135a‐3p and miR‐196a‐5p and the changes in the expression of 15 other miRs that have never been previously associated with human stroke. The identified miRs target processes and signaling pathways that play an important role in stroke progression and recovery, such as cell apoptosis, inflammation, oxidative stress, Wnt and MAPK signaling pathways, and neurogenesis.

### 1.3 Apoptosis

During the stroke, cells in the penumbra undergo apoptosis. The cells show shrinkage and blebbing, nuclear membrane breakdown, chromatin fragmentation, and the formation of apoptotic bodies at the edge of the cell surface (Fricker et al. 2018). There are two pathways of apoptosis: intrinsic and extrinsic. The intrinsic pathway also called mitochondrial pathway includes: the release of cytochrome c from mitochondria into the cytosol, the formation of apoptosomes from cytochrome c, protein Apaf‐1, and procaspase‐9, and activation of caspase‐9 which is followed by activation of caspase‐3 and proteases and nucleases that destroys the cells. In this pathway are also involved pro‐apoptotic proteins such as Bak, Bax, Bad, and Bcl‐XS that can heterodimerize with tBID and anti‐apoptotic proteins, Bcl‐2 or Bcl‐XL (Broughton et al. 2009; Uzdensky 2019). The extrinsic pathway starts with the binding of FasL to the Fas receptor. This leads to the recruitment of the Fas‐associated death domain protein (FADD) that contains a “death effector domain” that binds to procaspase‐8. The complex (FasL–Fas–FADD–procaspase‐8) called death‐inducing signaling complex (DISC) is responsible for the transactivation of procaspase‐8 to generate caspase‐8. Once activated, caspase‐8, activates caspase‐3 and induces PARP cleavage which results in DNA damage and activation of apoptosis (Broughton et al. 2009).

Many recent papers demonstrated the changes in the expression of different miRs that are involved in the regulation of apoptosis during the stroke. For example, miR‐99a and miR‐let‐7c‐5p, which target cleaved caspase‐3, are downregulated in the plasma of stroke patients and in the brain of mice after MCAO (Ni et al. 2015; Tao et al. 2015). The upregulation of miR‐25 inhibited cerebral I/R injury‐induced apoptosis through the downregulation of Fas/FasL (Zhang et al. 2016). Sun et al. (2013) showed that the upregulation of miR‐124 protected neurons against OGD‐induced cell death by increasing levels of anti‐apoptotic proteins such as Bcl‐2 and Bcl‐xl (Sun et al. 2013). Also, other miRNAs such as miR‐24 (Liu et al. 2016), miR‐134 (Huang et al. 2015) or miR‐29b (Ma et al. 2022) targeting caspase‐3 and Bcl‐xL, Bcl‐2, or Bax, are involved in stroke pathology. A very recent paper demonstrated that overexpression of miR‐122 resulted in decreased apoptosis, reduced cleaved caspase‐3 expression, and increased cell viability in astrocytes and HT22 cells subjected to OGD/R (Yu et al. 2024). Edwardson et al. (2023) showed that miR‐99a‐5p, miR‐127‐3p, miR‐128‐3p, miR‐181a‐3p, miR‐181a‐5p, miR‐382‐5p, miR‐433‐3p, miR‐491‐5p, and miR‐495‐3p are directly involved in the regulation of apoptosis in patients 15 and 30 days after the ischemic episode (Edwardson et al. 2023). All these studies suggest that miRNAs may play a crucial role in the regulation of programmed cell death during a stroke.

### 1.4 Inflammation

It is well known that inflammation is an important factor throughout all stages of ischemic stroke. Immediately after the stroke, there is a rapid activation of the peripheral immune system characterized by upregulation of proinflammatory cytokines such as IL‐1β, IL‐6, TNF‐α, and IFN‐c, as well as chemokines, C‐C motif chemokine ligand (CXCL)‐1 and CXCL‐2 (Simats and Liesz 2022). In humans, high circulating levels of TNF‐α and IL‐6 within the first 12 h after stroke are positively correlated with stroke severity and an unfavorable prognosis of stroke patients (Aref et al. 2020; Zaremba and Losy 2001).

Apart from the activation of peripheral inflammation, brain inflammation also peaks within 24–48 h after stroke. The damage‐associated molecular patterns (DAMPs) released by dying cells trigger microglial activation (Shi et al. 2019). The activated microglia release cytokines such as IL‐1β, TNF‐α, or IL‐6 and secrete extracellular matrix metalloproteinases (MMPs) that in turn increase the permeability of the blood–brain barrier (BBB) (Cao et al. 2023). Moreover, brain ischemia is followed by the astrocyte activation (reactive gliosis) characterized by increased glial fibrillary acidic protein (GFAP) expression, among others (Pekny and Pekna 2016). In addition, a recent study points to a crucial role of the NOD‐like receptor family pyrin domain‐containing 3 (NLRP3) inflammasome in the inflammatory damage following ischemia. The NLRP3 inflammasome consists of the cytosolic sensor molecule NLRP3, an adaptor protein apoptosis‐associated speck‐like protein containing a caspase activation recruitment domain (ASC), and a cysteine protease procaspase‐1. Upon assembly, caspase‐1 is activated, which in turn provokes the secretion of the proinflammatory cytokines IL‐1β and IL‐18, and it may also induce pyroptosis (Song et al. 2017). Recent data showed that post‐ischemic NLRP3 inhibition leads to the reduction of infarct volume, neurovascular unit protection, and BBB stabilization (Palomino‐Antolin et al. 2022).

It was found that miRNAs play an important role in slowing down ischemia‐induced neuroinflammation by diminishing the proinflammatory mediators. Recently it has been shown that intracerebroventricular (ICV) administration of miRNA‐192‐5p before MCAO attenuated acute cerebral injury by suppressing neuroinflammation in mice (He et al. 2024). MiR‐424 overexpression diminished ischemic brain injury through suppressing microglia activation (Zhao et al. 2013). The latest bioinformatics analysis identified hsa‐miR‐877‐5p as a key regulatory miRNA in acute ischemic stroke that can modulate immune and inflammatory responses via targeting IL‐23, CXCR3, and TNF‐α genes (Zhang et al. 2024). Many reviews on the role of different miRNAs in ischemia‐induced brain inflammation in vitro and in vivo were recently published (Elballal et al. 2024; Lian et al. 2021; Todoran et al. 2023; Yang et al. 2023). These confirm that miRNAs are important targets for future stroke therapies.

### 1.5 Pyroptosis

Pyroptosis is a novel programmed cell death that is caspase‐dependent cleavage of downstream effector protein gasdermins (GSDMs), inducing cell lysis and IL‐1β/IL‐18 secretion. The key events of pyroptosis include cell swelling, generation of membrane pores, loss of plasma membrane integrity, secretion of inflammatory factors, and chromatin condensation (Yu et al. 2021). There are two pathways of pyroptosis: the canonical and noncanonical pathway. In the canonical pathway, secretion of DAMPs and pathogen‐associated molecular patterns (PAMPs) from necrotic cells activates inflammasomes that are initiated to assemble. The activated inflammasomes cleave procaspase‐1 into caspase‐1, which in turn cleaves pro‐IL‐1β and pro‐IL‐18 to the mature forms. Secretion of IL‐1β and IL‐18 exacerbates the inflammatory response. The noncanonical inflammasome pathway is initiated by the binding of lipopolysaccharide (LPS) of Gram‐negative bacteria directly onto caspase‐4/5 in humans and caspase‐11 in murine. The binding of LPS onto these caspases promotes their oligomerization and activation. These caspases cleave GSDMD to release GSDMD‐N and trigger pyroptosis. In addition, an influx of potassium ions upon membrane permeabilization triggers activation of NLRP3, which then leads to the formation of NLRP3 inflammasome and activation of caspase‐1. These processes facilitate the cleavage of GSDMD and promote the maturation and release of proinflammatory cytokines (Wei et al. 2022).

It has been demonstrated that after ischemic stroke microglia, astrocytes, neurons, and brain microvascular endothelial cells (BMECs) undergo pyroptosis, which increases the breakdown of the BBB and thus aggravates brain injury (Long et al. 2023). Recent data showed an involvement of different miRNAs in the regulation of pyroptosis after ischemic stroke. It has been demonstrated that ICV injection of agomirs miR‐96‐5p or miR‐124 alleviates brain injury and improves neurological impairment after MCAO in rodents via inhibition of pyroptosis (Jin et al. 2024; Sun et al. 2020). Furthermore, miRNA‐124 inhibited STAT3 activation, which also decreased pyroptosis (Sun et al. 2020). In the cellular model of ischemia, miR‐139 protected SH‐SY5Y cells against OGD/R‐induced cell pyroptosis by inhibition of NLRP3 inflammasome activation (Wang, Luo, et al. 2020). In turn, the knockdown of miR‐155‐5p inhibited the inflammation response and cell pyroptosis of MCAO rats via modulating DUSP14/TXNIP/NLRP3 pathway (Shi et al. 2022).

### 1.6 Ferroptosis

Ferroptosis is defined as an iron‐dependent form of cell death characterized by oxidative damage to cells caused by the accumulation of lipid peroxides (Jhelum and David 2022). The primary mechanism of ferroptosis involves the catalysis of the highly expressed polyunsaturated fatty acids (PUFAs) on the cell membrane to undergo lipid peroxidation under the action of Fe^2+^ or ester oxygenase, thereby inducing cell death (Forcina and Dixon 2019). To initiate lipid peroxidation on cell membranes, acyl‐CoA synthetase long‐chain family member 4 (ACSL4) first catalyzes the conversion of arachidonic acid (AA) into AA‐CoA, followed by the esterification of AA‐CoA into phosphatidylethanolamines (PE) with the involvement of lysophosphatidylcholine acyltransferase 3 (LPCAT3). In addition, lipoxygenases (LOXs) catalyze the oxidation of excessive AA‐CoA to HETE, such as 5‐HETE, which also contributes to ferroptosis. It is important to underline that ACSL4 cannot be replaced by other members of the ACSL family, and its overexpression promotes the sensitivity of cells to ferroptosis (Ding et al. 2023). Moreover, stroke induces a dysfunction in an amino acid antiporter (System Xc−) mediating the exchange of extracellular l‐cystine and intracellular l‐glutamate across the plasma membrane. This leads to a deficiency in intracellular cystine, depletion of GSH, and reduction in GPX4 activity, thereby increasing ROS in lipids and inducing cell ferroptosis (Forcina and Dixon 2019). Experimental data confirm the activation of ferroptosis after MCAO in murine brains (Liang et al. 2022; Tuo et al. 2022). Moreover, N27 neuronal cells subjected to OGD for 2 h have had a raised level of intracellular Fe^2+^, lipid peroxides, lipid hydroperoxides, and ROS (Tuo et al. 2022). Recently published studies point to an involvement of miRNAs in the regulation of ferroptosis after the stroke. It has been shown that the expression level of miR‐27a was increased and ferroptosis was aggravated in rat brain tissue after MCAO followed by reperfusion. These effects were reversed with antagomiR‐27a administration (Zhu et al. 2023). MiR‐214 overexpression significantly suppressed ferroptosis, possibly through reducing lipid peroxidation and iron deposition in in vivo and in vitro models of brain ischemia (Lu et al. 2020). The above‐cited studies suggest that exploring the molecular mechanism related to ferroptosis could be a useful tool for future stroke therapies.

### 1.7 Oxidative Stress

Due to the presence of high amounts of unsaturated lipids, low levels of antioxidants, and high oxygen consumption, the brain is the main target for oxidative stress (Cobley et al. 2018). After reperfusion, oxidative stress is caused by excessive ROS generation by mitochondria, but also through nicotinamide adenine dinucleotide phosphate (NADPH) oxidase and xanthine oxidase (XO) enzymes (Briones‐Valdivieso et al. 2024).

During the stroke, the increase in intracellular Ca^2+^ level activates different enzymes such as calmodulin or protein kinase C (PKC). Calmodulin stimulates neuronal nitric oxide synthase (NOS) activity. Nitric oxide (NO) produced by NOS reacts with superoxide anions and forms reactive nitrogen species called peroxynitrite (Förstermann and Sessa 2012, Wang, Hong, and Yang 2022). Protein Kinase C increases the activity of NAPDH oxidase, which in turn generates additional ROS (Cosentino‐Gomes et al. 2012). Moreover, activated microglia and peripheral macrophages generate NO via inducible NOS. Increased BBB permeability facilitates the penetration of peripheral immune cells into the brain where they contribute to oxidative damage via myeloperoxidase. NADPH oxidase also contributes to ROS production in macrophages and neutrophils. The excessive ROS formation and downregulation of antioxidant enzymes during the stroke leads to the destruction of intracellular lipids, proteins, and nucleic acids followed by mitochondrial swelling, cell injury, and death (Canton et al. 2021; Cherubini et al. 2000; Wang, Lu, et al. 2022). Therefore, it is important to inhibit the cascade of oxidative stress and prevent the cell's death. One of the strategies could be a modulation of the expression of miRNA that targets genes involved in antioxidant protection. Indeed, Ma et al. (2023) showed that the miR‐9a‐5p could alleviate oxidative stress in SH‐SY5Y cells after OGD/R injury via increasing SOD and decreasing MDA levels, which in turn inhibits ROS formation (Ma et al. 2023). Another study demonstrated that inhibition of miR‐423‐5p attenuates ischemia‐induced oxidative stress via modulation of protein expression levels of MDA, GSH, and SOD (Luo et al. 2022). Also, inhibition of miR‐29b suppressed oxidative stress in ischemic stroke via upregulation of SOD level in MCAO rats (Ma et al. 2022). Instead, inhibition of miR‐9‐3p protected rats against cerebral I/R injury through activating GSK‐3β/Nrf2/ARE signaling (Zhou, Yang, et al. 2021). Furthermore, miR‐454 seems to play an important role in modulation of oxidative stress. The miR‐454 agomir declined NADPH oxidase 4 (NOX4) level and ROS production in rats suffering from I/R (Zhang et al. 2022). These are only some examples from many others already published that point to a key role of miRs in the regulation of oxidative stress during ischemic stroke.

### 1.8 Other Signaling Pathways Involved in Mechanisms of Stroke

#### 1.8.1 AMP‐Activated Protein Kinase (AMPK)

AMPK belongs to the serine/threonine (Ser/Thr) kinase group and is known as an energy sensor. Under ischemic conditions, AMPK is activated by increases in AMP/ATP or ADP/ATP ratios, where the increase in AMP is always much greater than the increase in ADP or decrease in ATP. In addition to AMP, liver kinase B1 (LKB1), Ca2+/calmodulin‐dependent protein kinase β (CaMKKβ), 5‐aminoimidazole‐4‐carboxamide ribonucleoside (AICAR), and antimycin A are able to activate AMPK (Jiang et al. 2018). For example, CaMKKβ can activate AMPK in response to increases in cellular Ca2+ without any significant change in ATP/ADP/AMP levels (Kim et al. 2016). It has been shown that MCAO increased the activity of LKB1 in mice, which was followed by increased AMPK expression (Li et al. 2007). Recently, Wang, Yuan, et al. (2019) demonstrated that inhibition of miRNA‐27b enhances neurogenesis via AMPK activation in a mouse ischemic stroke model (Wang, Yuan, et al. 2019). Moreover, activation of AMPK induced a robust expression of miR‐210‐3p both in vitro in primary cortical neurons and following ischemic stroke in vivo (Pfeiffer et al. 2021).

#### 1.8.2 Wnt Signaling Pathways

Wnt signaling plays a crucial role in CNS development, particularly in the establishment and maintenance of synaptic structure and neuronal functions. There are at least two Wnt pathways recognized till now: the canonical and non‐canonical pathways (Mo et al. 2022). The canonical Wnt signaling pathway, known as the Wnt/β‐catenin signaling, starts with Wnt protein binding to the transmembrane receptor frizzled protein (Fz) and co‐receptor low‐density lipoprotein receptor‐related protein (LRP5/6). This in turn activates a series of biochemical processes leading to the nuclear translocation of β‐catenin. Within the nucleus, β‐catenin assembles with transcription factor T cell factor (TCF)/lymphoid enhancing factor (LEF) and activates the transcription of target genes such as TCF/LEF, cyclin D1, and survivins (Nelson and Nusse 2004). In the ischemic brain, the activity of the Wnt/β‐catenin signaling pathway decreases (Mo et al. 2022). It has been shown that miRs can be involved in the regulation of the Wnt signaling pathway in ischemic stroke. For example, Wang et al. (2017) showed that miR‐148b regulates the proliferation and differentiation of neural stem cells via Wnt/β‐catenin signaling in the rats undergoing the MCAO procedure (Wang et al. 2017). A more recent study demonstrated that miR‐34 inhibits the Wnt/β‐catenin pathway by targeting Wnt1 in an MCAO rat model, and this can be reverted by electroacupuncture (Cao et al. 2021).

### 1.9 Phytochemicals

Phytochemicals, bioactive compounds derived from plants, have gained substantial interest for their potential therapeutic benefits in various medical conditions, including cerebral ischemia (Xu et al. 2021). These compounds, which include a diverse array of molecules such as flavonoids, alkaloids, and terpenoids, are renowned for their antioxidant, anti‐inflammatory, and neuroprotective properties (Grosso et al. 2023). Increasingly, research has focused on the ability of phytochemicals to modulate miRNAs—small non‐coding RNAs critical in gene regulation and implicated in the pathophysiology of cerebral ischemia (Neag et al. 2022). Evidence indicates that certain phytochemicals can regulate miRNA expression, thereby impacting crucial cellular processes such as apoptosis, inflammation, and oxidative stress, which are central to the progression and recovery from cerebral ischemia (Ferrero et al. 2021; Kang 2019; Lee and Im 2021). For instance, curcumin, a polyphenol derived from turmeric, has been shown to regulate the miR‐1287‐5p/LONP2 axis and miR‐7/RelA p65 axis in an OGD/R model, thereby providing neuroprotection by inhibiting oxidative stress (Fan and Lei 2022). Similarly, resveratrol, a stilbenoid found in grapes, downregulates miR‐155 to attenuate neuroinflammation and enhance cell survival under ischemic conditions (Wang, Yu, and Wu 2022). Among the phytochemicals studied, flavonoids including quercetin or epigallocatechin gallate (EGCG) have shown promising results. Quercetin, present in many fruits and vegetables, modulates miR‐21, which reduces inflammation and apoptosis in ischemic brain tissue (Wang, Fu, et al. 2022). EGCG, a major component of green tea, enhances the expression of miR‐210, promoting angiogenesis and cell survival under hypoxic conditions (Wang et al. 2011). These findings suggest that flavonoids exert multifaceted protective effects in cerebral ischemia through miRNA modulation. Alkaloids, another significant class of phytochemicals, also exhibit neuroprotective properties. Berberine, an isoquinoline alkaloid, regulates the miR‐188/nitric oxide synthase 1 (NOS1) pathway, promoting cell viability and attenuating Aβ‐induced neuronal damage in Alzheimer's disease (Cheng et al. 2022). The effect of berberine preserves mitochondrial function and prevents cell death, highlighting its potential as neuroprotection. Terpenoids, including ginsenosides from ginseng, are similarly implicated in miRNA modulation related to cerebral ischemia. Ginsenoside Rd regulates the miR‐139‐5p/FoxO1/Keap1/Nrf2 axis, playing a critical role in attenuating the anti‐pyroptotic response and mitigating cerebral ischemia/reperfusion injury (Zhao et al. 2022). This regulatory effect on miRNA expression underscores the potential of ginsenosides to facilitate recovery and repair processes in the brain.

Exploring these natural compounds and their interactions with miRNAs stresses their potential in developing novel and safer therapeutic approaches to mitigate the effects of cerebral ischemia. This review systematically examines current evidence from both in vitro and in vivo studies on the modulation of miRNAs by phytochemicals, providing insights into their mechanisms of action and therapeutic potential in stroke treatment. Understanding the molecular interactions between phytochemicals and miRNAs enhances our appreciation of their role in neuroprotection and paves the way for innovative approaches to improve brain recovery post‐ischemia.

## 2 Materials and Methods

### 2.1 Eligibility Criteria

Table 1 reports the PICO (Population, Intervention, Comparison, Outcome) format to provide a more detailed explanation of eligibility criteria.

**TABLE 1:** Description of the PICO strategy.

Parameter	Eligibility criteria
Population (P)	Any animal or cellular model of brain hypoxia/ischemia
Intervention (I)	Any dietary bioactive compound (e.g., polyphenols, carotenoids), provided as extract and pure compounds.
Comparison (C)	Control group without bioactive compound.
Outcome (O)	Changes in miRNA expression related to inflammatory, cell death and oxidative stress pathways

### 2.2 Search Strategy

The current systematic review has been carried out in accordance with the Preferred Reporting Items for Systematic Reviews and Meta‐Analyses (PRISMA) guidelines (Page et al. 2021).

A systematic search was performed in the three databases: Embase, Scopus, and PubMed. The following search terms were used: ((polyphenol) OR (glucosinolate) OR (carotenoid) OR (anthocyanin) OR (flavonoid) OR (flavanol) OR (flavone) OR (flavanone) OR (flavanol) OR (isoflavone) OR (anthocyanin) OR (phenolic AND acid) OR (stilbene) OR (lignan) OR (curcumin) OR (tannin) OR (rosmarinic AND acid) OR (resorcinol) OR (hydroxytyrosol) OR (isothiocyanate) OR (diindolylmethane) OR (indole‐3 AND carbinol)) AND ((ischemia) OR (ischemic AND stroke) OR (stroke) OR (infarction) OR (hypoxia) OR (embolic) OR (thrombotic)) AND ((mirna) OR (microrna)).

### 2.3 Data Selection and Data Extraction

Primary literature search results were evaluated by two investigators (JR. and BM.). All articles were scrutinized by their titles and abstracts and selected based on the predetermined inclusion criteria. Subsequently, all the selected articles underwent a full‐text analysis by both authors, taking into consideration the established criteria for inclusion and exclusion. The discrepancies in the evaluation of records inclusion were assessed by a third independent reviewer (M.M.).

### 2.4 Risk of Bias for Quality Assessment of Preclinical Studies

The risk of bias of the included studies was assessed using SYRCLE's RoB criteria for in vivo studies (Hooijmans et al. 2014) and a modified version for in vitro studies (see Table S1, de la Rosa González et al. 2024). Each study was evaluated based on 10 domains of risk of bias. The responses for each domain were classified as “YES,” indicating low risk, “NO,” representing high risk, and “UNCLEAR” when the risk could not be determined. Data analysis was performed using Python (version 3.11) to organize and visualize the results. The data were summarized in two types of graphical representations. The first consists of a heatmap, which depicts the risk of bias for each combination of study and question through a color‐coded matrix. Green indicates low risk (“YES”), yellow represents an undetermined risk (“UNCLEAR”), and red indicates high risk (“NO”). The second representation comprises a stacked horizontal bar chart, where the risk categories are displayed based on the number of studies falling into each category for each question, using the same color scheme as the heatmap. The figures were generated separately for in vivo and in vitro studies. The data were normalized and organized into matrices to ensure consistency in visualization. The analyses and figure generation were carried out utilizing Python libraries such as Pandas for data management, Seaborn for heatmaps, and Matplotlib for the stacked bar charts.

To analyze the quality of the data in the articles included in this review, SYRCLE's RoB tool was used separately by two investigators (JR and MM); any possibility of disagreement during the process was solved by reaching a consensus or by a third investigator (LC).

## 3 Results

### 3.1 Literature Search

**FIGURE 1:**
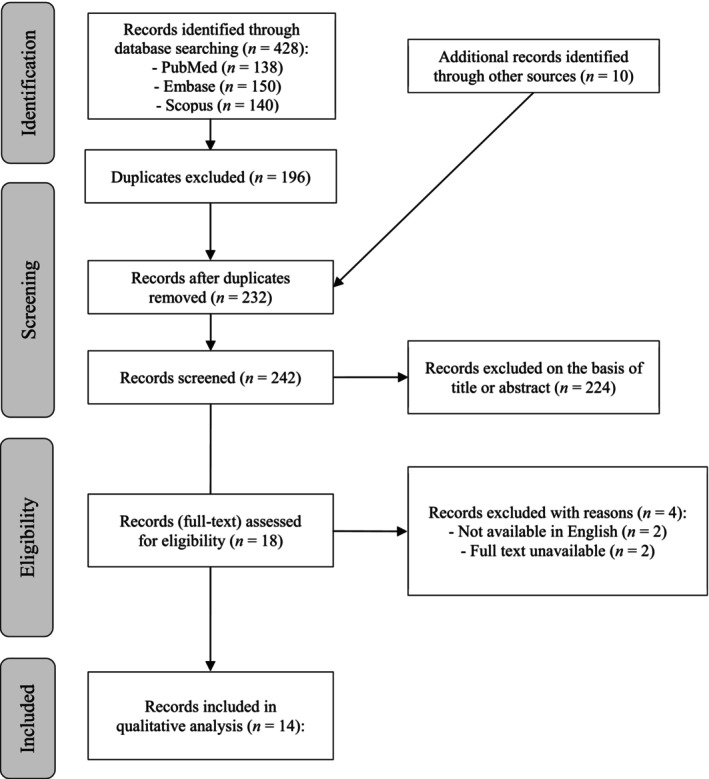
PRISMA flowchart of studies' selection process.

The bibliographic search in PubMed, Scopus, and Embase databases produced a total of 428 papers. Figure 1 illustrates the flow diagram of the selection process for this systematic review. Initially, 196 papers were excluded due to duplication. The remaining 232 articles were supplemented with 10 additional articles identified through other sources. From these 242 articles, 224 were considered irrelevant based on title and/or abstract information. Furthermore, four articles were excluded since no English or full‐text versions were available. Finally, 14 articles were included in this review.

### 3.2 Study Characteristics

#### 3.2.1 In Vitro Studies

The main characteristics of the selected studies in vitro are summarized in Table 2. This table outlines the type of cells and methods used to induce in vitro brain ischemia, phytochemicals in different concentrations, type (co‐, pre‐, or post‐treatment) and duration of the treatment, changes in miRNA expression, and molecular pathways involved in neuronal cell protection. Of the 14 articles, nine studied the effects of phytochemicals in cellular models of brain ischemia, with six using cell lines (i.e., PC‐12, HT22, SK‐N‐SH cells) and three using rat or mouse primary neuronal cell cultures or neuronal stem cells. In all studies, phytochemicals exerted changes in miRNA expression, followed by inhibition of ischemia‐induced apoptosis, inflammation, oxidative stress, pyroptosis, or ferroptosis.

**TABLE 2:** Overview of in vitro studies with the use of phytochemicals that evoke changes in miRs expression to protect neuronal cells against ischemia‐induced damage.

Publication	Type of cell culture	Duration of hypoxia/ischemia in vitro	Phytochemical	Concentration	Type and treatment duration	miR‐regulated	Target genes/proteins and signaling pathways
(Dai et al. 2024)	SK‐ N‐ SH cells	OGD 8 h/24 h Reox	Baicalin	10 μM	24 h pre‐treatment	↑miR‐556‐3p	Baicalin inhibited expression of ACSL4, decreased expression of IL‐6, IL‐1β, and TNF‐α, and levels of Fe^2+^, ROS, MDA
(Liang and Sun 2023)	HT22 cells	OGD 3 h/24 h Reox	Curcumin	20, 40, 60 μg/g	Not indicated	↓miR‐145‐5p	Curcumin inhibited TNF‐α, IL‐6, IL‐10 protein levels, decreased MDA level, and increased CAT and SOD levels
(Shi et al. 2022)	Rat primary hippocampal cells	OGD/Reox	Puerarin	80 μM	0, 24, 48 h post‐treatment	↑miR‐223‐3p	Puerarin downregulated cleaved caspase‐3 and Bax, NLRP3, ASC and upregulated Cyclin D1 and CDK2
(Wang, Wan, et al. 2020)	Rat primary cortical cells	OGD 4 h/20 h Reox	Safflor Yellow B (SYB)	0.5 mM	24 h pre‐treatment	↓miR‐134	SYB promoted CREB activation, decreased NOX4 level, increased Nrf2 expression
(T. Zhang et al. 2021)	SH‐SY5Y cells	OGD 6‐36 h/48 h Reox	Curcumin	25 μM	24 h pre‐treatment	↑miR‐1287‐5p	Curcumin decreased LONP2, Bad, cleaved caspase 3, and Cyt‐C protein levels, increased Bcl‐2 protein level and inhibited ROS formation
(Li et al. 2019)	Rat primary neuronal stem cells	OGD 3 h/48 h Reox	Theaflavin	2,10 μM	2 h pre‐treatment	↓ miR‐128‐3p	Theaflavin upregulated Nrf2 pathway, inhibited ROS formation, decreased level of MDA and increased the level of SOD and GSH‐Px
(Wang, Ma, and Yang 2019)	Rat hippocampal stem cells	Hypoxia 8 h	Puerarin	60 μM	24 h pre‐treatment	↑ miR‐214	Puerarin decreased the protein levels of cleaved caspase‐3 and caspase‐9, and increased protein levels of phosphorylated forms of PI3K, AKT, MEK, and ERK
(Yan et al. 2019)	PC‐12	Hypoxia 24 h	Quercetin	10 μM	24 h treatment	↓ miR‐122	Quercetin activated AMPK and Wnt/β‐catenin signaling pathways
(Bian et al. 2017)	PC‐12	OGD 3 h/24 h Reox	Resveratrol	10 μM	1 h pre‐treatment	↑ miR‐96	Resveratrol decreased mRNA and protein level of Bax

Abbreviations: OGD, oxygen and glucose deprivation; Reox (R), reoxygenation.

#### 3.2.2 The Effect of Polyphenols on miRs Modulation

Flavonoids such as baicalin, Safflor yellow B (SYB) and quercetin were administered 24 h before (Dai et al. 2024; Wang, Wan, et al. 2020) or during hypoxic/ischemic stimuli (Yan et al. 2019). The 8 h OGD followed by 24 h of reoxygenation decreased the expression of miR‐556‐3p in the human neuroblastoma cell line (SK‐N‐SH). Baicalin significantly increased miR‐556‐3p expression, which was followed by the inhibition of apoptosis, cell proliferation, oxidative stress, inflammation, and ferroptosis (Dai et al. 2024). In rat primary hippocampal cell cultures, 4 h OGD followed by 20 h of reoxygenation increased miR‐134 expression. Safflor yellow B, a component of Safflower yellow‐flavonoid extracted from *Carthamus tinctorius* L., was able to prevent OGD/R‐induced miR‐134 reduction and subsequently activated CREB. This led to increased antioxidant capacities, improved neuronal cell respiration, and reduced apoptosis (Wang, Wan, et al. 2020). It has also been demonstrated that 24 h hypoxia increased the expression of miR‐122 in PC‐12 cells, and quercetin was able to decrease miR‐122 expression, which was accompanied by activations of AMPK and Wnt/β‐catenin signaling pathways (Yan et al. 2019).

Pre‐treatment of neuronal cells with polyphenols such as curcumin (Liang and Sun 2023; Zhang et al. 2021), theaflavin (Li et al. 2019) or resveratrol resulted in great neuroprotection against OGD/R‐induced cell death (Bian et al. 2017). In two different cell lines, that is., SH‐SY5Y and HT22, curcumin was able to revert OGD/R‐induced changes in miRs expression. In SH‐SY5Y, curcumin decreased OGD/R‐stimulated expression of miR‐145‐5p and inhibited OGD‐induced inflammation and oxidative stress (Liang and Sun 2023). Zhang et al. (2021) demonstrated that curcumin was also able to upregulate OGD/R‐downregulated miR‐1287‐5p expression, and this was followed by inhibition of apoptosis (Zhang et al. 2021). Two hours of pre‐treatment with theaflavin—flavonoid derived from black tea—protected primary rat neuronal stem cells (NSCs) from OGD/R‐induced damage via decreasing the expression level of miR‐128‐3p. One of the targets of miR‐128‐3p is the *Nrf2* gene—which is involved in antioxidant defense. It has been shown that theaflavin decreased miRNA‐128‐3p expression that was previously induced by OGD/R in NSCs. This was accompanied by an increase in *Nrf2* gene expression and oxidative stress reduction (Li et al. 2019). Resveratrol is a natural stilbene present at relatively high concentrations in grape skin, seeds, and red wine. It has been shown that 1 h pre‐treatment of the PC‐12 cells with resveratrol prevented OGD/R‐induced cell apoptosis via upregulation of miR‐96, which in turn decreased the expression of Bax (Bian et al. 2017).

**FIGURE 2:**
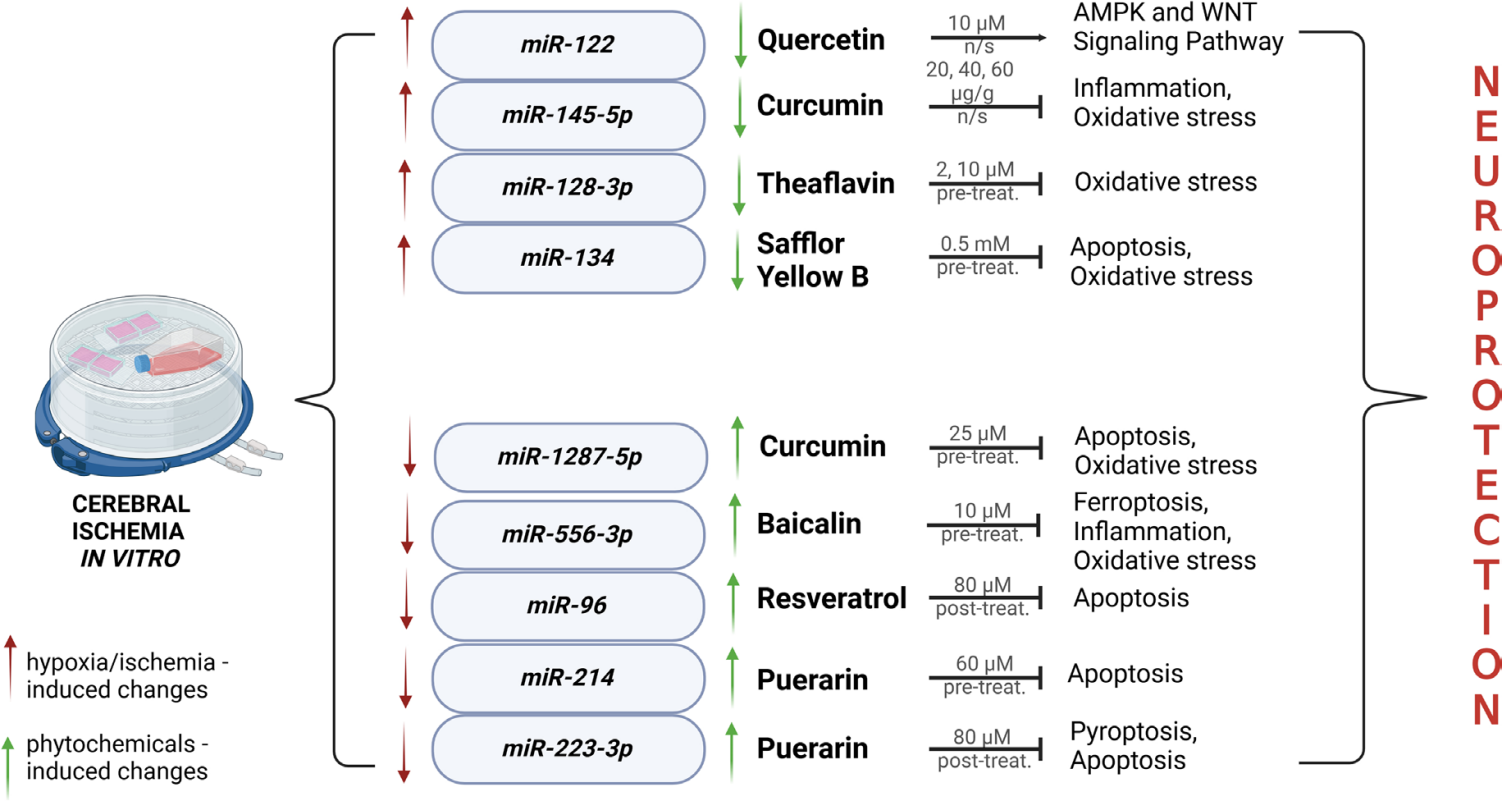
Schematic model representing the changes in miRs expression evoked by hypoxia/ischemia in vitro and phytochemicals.

Puerarin is a bioactive isoflavone‐C‐glucoside, extracted from the dried root of Pueraria labata (Willd.) Ohwi. Shi et al. (2022) demonstrated that treatment of rat primary hippocampal cells with Puerarin inhibited OGD/R induced pyroptosis by miR‐223‐3p upregulation, which in turn decreased NLRP3 expression level (Shi et al. 2022). In primary rat hippocampal stem cells subjected to 8 h of hypoxia, 24 h pre‐treatment with Puerarin increased expression of miR‐214, which was accompanied by inhibition of apoptosis and activation of prosurvival signaling pathways like PI3K/AKT and MEK/ERK (Wang, Ma, and Yang 2019) (Figure 2).

#### 3.2.3 In Vivo Studies

The main characteristics of the studies in vivo are presented in Table 3. This table includes the strain of animals, methods used to induce in vivo brain ischemia, phytochemical characterization and doses administered, type (pre‐ or post‐treatment) and duration of the treatment, method of injection, changes in miRNA expression, and molecular pathways involved in brain protection. Of the 14 articles, nine studied the effects of phytochemicals in in vivo models of brain ischemia, primarily using rats (7 out of 9) and, in two cases, mice. Most studies induced brain damage using the middle cerebral artery occlusion (MCAO, with or without reperfusion). In two articles, the ischemia was induced by ligation of the left carotid artery followed by hypoxia, which is a model of perinatal asphyxia. In all the studies, ischemia evoked changes in miRs expression, and phytochemicals were able to revert them, inhibiting ischemia‐induced apoptosis, inflammation, oxidative stress, pyroptosis, or ferroptosis.

**TABLE 3:** Overview of in vivo studies with the use of phytochemicals that evoke changes in miRNAs expression protecting the brain against ischemia‐induced damage.

Publication	Strain of experimental animals	In vivo brain ischemia duration	Phytochemical	Dose	Treatment duration and route	miR‐regulated	Target genes/proteins and signaling pathways
(Dai et al. 2024)	C57BL/6J mice	tMCAO 1 h followed by 24 h reperfusion	Baicalin	100 mg/kg	IV injection	↑ miR‐556‐3p	Baicalin decreased protein level of ACSL4
(Liu et al. 2023)	Rats	tMCAO 2 h followed by 24 h reperfusion	Resveratrol	60 μg/g	3 days before MCAO by IP injection	↑ miR‐450b‐5p	Resveratrol inhibited KEAP1 expression and increased protein level of NRF2
(Shi et al. 2022)	Sprague–Dawley rats	tMCAO 90 min followed by 48 h reperfusion	Puerarin	100 mg/kg	30 min before MCAO and 8 h after reperfusion by IP injection	↑ miR‐223‐3p	Puerarin downregulated cleaved caspase‐3 Bax, NLRP3 and ASC and upregulated Cyclin D1 and CDK2
(Teertam et al. 2020)	Sprague–Dawley rats	tMCAO for 60 min	Resveratrol	20 mg/kg	30 min before MCAO by IP injection	↑ miR‐149‐5p	Resveratrol decreased p53 protein level, caspase‐3 activity, increased Sirt1 level
(Rzemieniec et al. 2020)	Wistar rat pups	Ligation of left common carotid artery for 60 min followed by 75 min hypoxia	3,3′‐diindolylmethane (DIM)	0.1, 10 or 100 mg/kg	24 h, 48, and 72 h after perinatal asphyxia by IP injection	↓ miR‐181b‐5p	DIM decreased cleaved caspase‐3 and caspase‐9 protein level
(Ma et al. 2020)	C57/BL mice	tMCAO for 120 min	Resveratrol	100 mg/kg	0 h, 8 h and 18 h after MCAO by IP injection	↓ miR‐155	Resveratrol increased CD206 and Arginase‐1 expression, decreased the expression of IL‐1 and IL‐6.
(Wang, Wan, et al. 2020)	Sprague–Dawley rats	tMCAO 1 h followed by 23 h of reperfusion	Safflor Yellow B	6 mg/kg	3 days before MCAO by IV injection	↓ miR‐134	SYB diminished ROS level, MDA relative content, NADPH oxidase activity, ratio Bax/Bcl‐2, caspase‐3 level and Nox4 expression. SYB increased SOD and GSH‐Px relative activity, Nrf2 expression and CREB level
(Li et al. 2019)	Sprague–Dawley rats	tMCAO for 90 min	Theaflavin	10, 50 mg/kg	2 h after MCAO and then once a day for 7 days by IV injection	↓ miR‐128‐3p	Theaflavin upregulated Nrf2 pathway, it inhibited ROS formation, decreased level of MDA and increased the level of SOD and GSH‐Px
(Bian et al. 2017)	Wistar rat pups	Ligation of left carotid artery followed by 2.5 h of hypoxia	Resveratrol	100 mg/kg	0 h, 8 h and 18 h post‐treatment by IP injection	↑ miR‐96	Resveratrol decreased mRNA and protein level of BAX
(Wang et al. 2014)	Sprague–Dawley rats	Focal ischemia 2 h followed by reperfusion	Calycosin	5,10,20 mg/kg	14 days before ischemia by IP injection	↑ miR‐375	Calycosin increased protein level of ERα, BCL‐2 and decreased RASD1

Abbreviations: IP, intraperitoneal; IV, intravenous; tMCAO, transient middle cerebral artery occlusion.

#### 3.2.4 The Effect of Polyphenols on miRs Modulation

Flavonoids such as baicalin and Safflor yellow B (SYB) were injected intravenously to mice or rats, respectively (Dai et al. 2024; Wang, Wan, et al. 2020). In both cases, to induce brain ischemia, the authors used tMCAO (1 h) followed by 23/24 h of reperfusion. Dai et al. (2024) showed that the tMCAO procedure decreased the expression of miR‐556‐3p, and the treatment with baicalin (100 mg/kg) was able to revert this change, concomitantly with decreasing the protein level of ACSL4 involved in ferroptosis (Dai et al. 2024). Treatment with baicalin decreased significantly the infarct volume and improved neurological score. These experiments confirmed the results in vitro described above. Similarly, Wang, Wan, et al. (2020) were able to confirm the results obtained in vitro. Indeed, they showed that the 3‐day pre‐treatment of rats with SYB, followed by tMCAO, decreased miR‐134 expression and inhibited ischemia‐induced brain inflammation, oxidative stress, and apoptosis (Wang, Wan, et al. 2020).

In accordance with the study in vitro, the treatment of rats with polyphenols such as theaflavin (Li et al. 2019) or resveratrol (Bian et al. 2017; Liu et al. 2023; Ma et al. 2020; Teertam et al. 2020) resulted in great neuroprotection against brain ischemia‐induced damage. A 3‐day pre‐treatment of rats with resveratrol (60 ug/g) improved tMCAO (2 h)–reperfusion (24 h)‐induced injury by promoting microglial M2 polarization. This was directly associated with increasing expression of miR‐450b‐5p, KEAP1 downregulation, and *Nrf2* upregulation (Liu et al. 2023). Resveratrol (20 mg/kg) injected intraperitoneally 30 min before tMCAO was able to rescue the brain against ischemic damage via upregulation of miR‐149‐5p and inhibition of apoptosis (Teertam et al. 2020). Similarly, to Liu et al. 2023, Ma et al. 2020 showed that resveratrol promotes M2 polarization and inhibits neuroinflammation; however, the mechanisms involved in these changes are different (Liu et al. 2023; Ma et al. 2020). Here, resveratrol (100 mg/kg) administered three times at 0 h, 8 h, and 18 h after tMCAO decreased expression of miR‐155, which was followed by increased expression of Arginase‐1 and CD206 and downregulation of Il‐6 and Il‐1. The same administration protocol of resveratrol (100 mg/kg) was used by Bian et al. 2017, who subjected rat pups to perinatal asphyxia. The authors showed that resveratrol was able to revert PA‐induced downregulation of miR‐96 in the hippocampus and cerebral cortex of the rats (Bian et al. 2017). This was accompanied by a decrease of pro‐apoptotic Bax mRNA expression and protein level. Treatment of rats with theaflavin (10 and 50 mg/kg) for 7 days after MCAO decreased the expression of miR‐128‐3p and upregulated *Nrf2* expression. Similarly, to the results in vitro, theaflavin in vivo reduced oxidative stress via modulation of the activity of the antioxidant enzymes (Li et al. 2019).

Isoflavones such as puerarin and calycosin exerted neuroprotective effects in rat models of brain ischemia. Intraperitoneal injection of puerarin (100 mg/kg) 30 min before and 8 h after tMCAO resulted in decreased neurological deficit score and infarcted area volume. This was accompanied by increased miR‐223‐3p level, downregulation of apoptosis, and NLRP3‐mediated pyroptotic cell death (Shi et al. 2022). Isoflavone calycosin is a phytoestrogen extracted from the Chinese medical herb Radix Astragali. It has been shown that the treatment of rats with calycosin in 3 different doses (5, 10, 20 mg/kg) for 14 days before induction of focal cerebral ischemia significantly reduced the infarcted volume and brain water content, and improved the neurological deficit. Calycosin downregulated RASD1 (Dexamethasone‐induced Ras‐related protein 1) protein level, and an upregulated expression of ER‐α, miR‐375, and anti‐apoptotic Bcl‐2. These findings demonstrate that calycosin exerts neuroprotective effects in cerebral ischemia/reperfusion rats by estrogen receptor activation, positive feedback regulation by miR‐375, deregulation of RASD1, and upregulation of Bcl‐2 (Wang et al. 2014).

**FIGURE 3:**
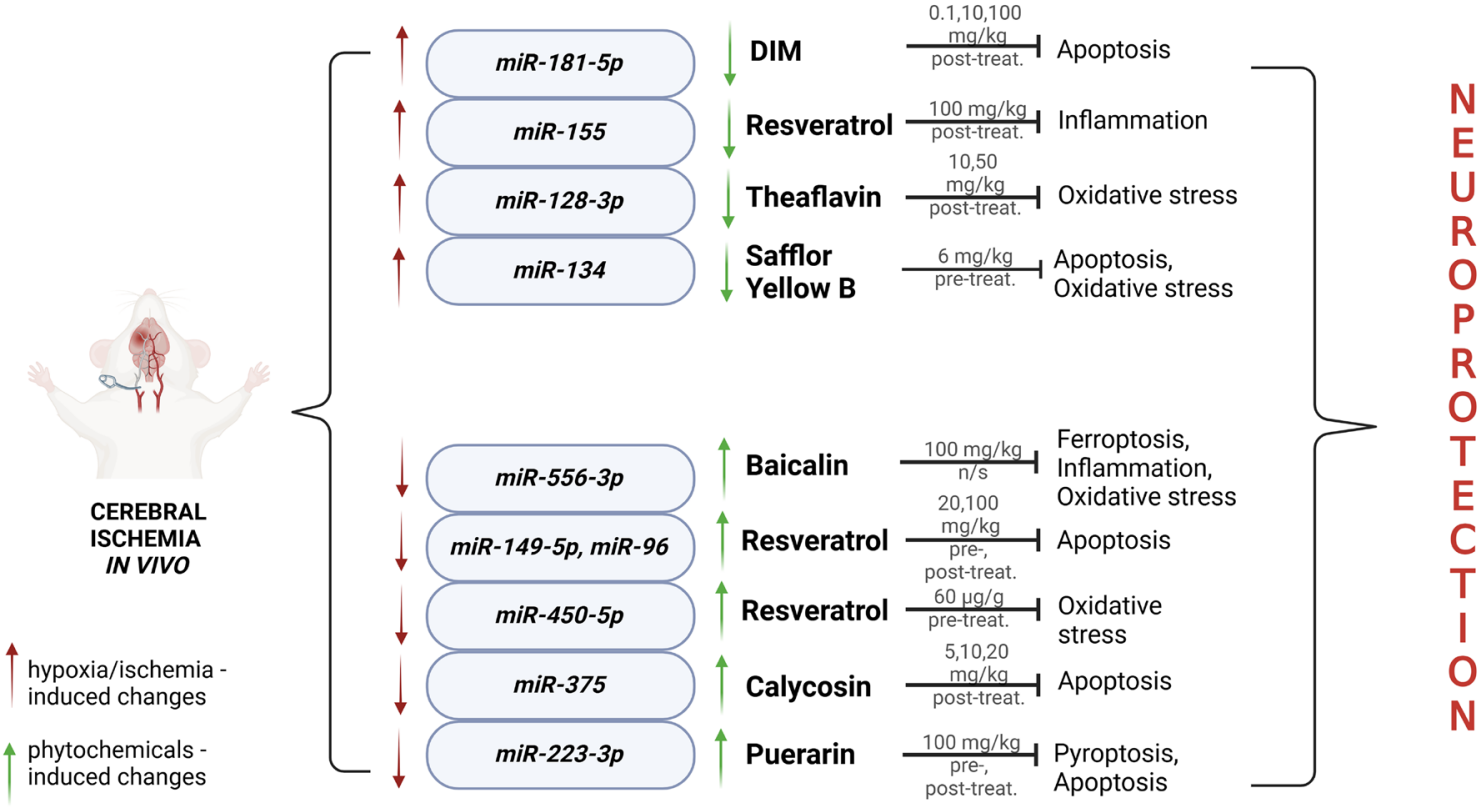
Schematic model representing the changes in miRs expression evoked by hypoxia/ischemia in vivo and phytochemicals.

Another phytoestrogen called 3,3′‐diindolylmethane (DIM), which is an active metabolite of indole‐3 carbinol found in cruciferous vegetables, exerted protective action in the rat model of perinatal asphyxia. It has been shown that the administration of DIM (0.1, 10, and 100 mg/kg) for three consecutive days after perinatal asphyxia (PA) restored the weight of the ipsilateral hemisphere and normalized cell number in the rat brain tissues after PA. DIM (10 mg/kg) downregulated the PA‐induced expression of miR‐181b, which was followed by inhibition of caspase‐dependent apoptosis (Rzemieniec et al. 2020) (Figure 3).

#### 3.2.5 The Classification of Phytochemicals Into the Classes and Subclasses

**FIGURE 4:**
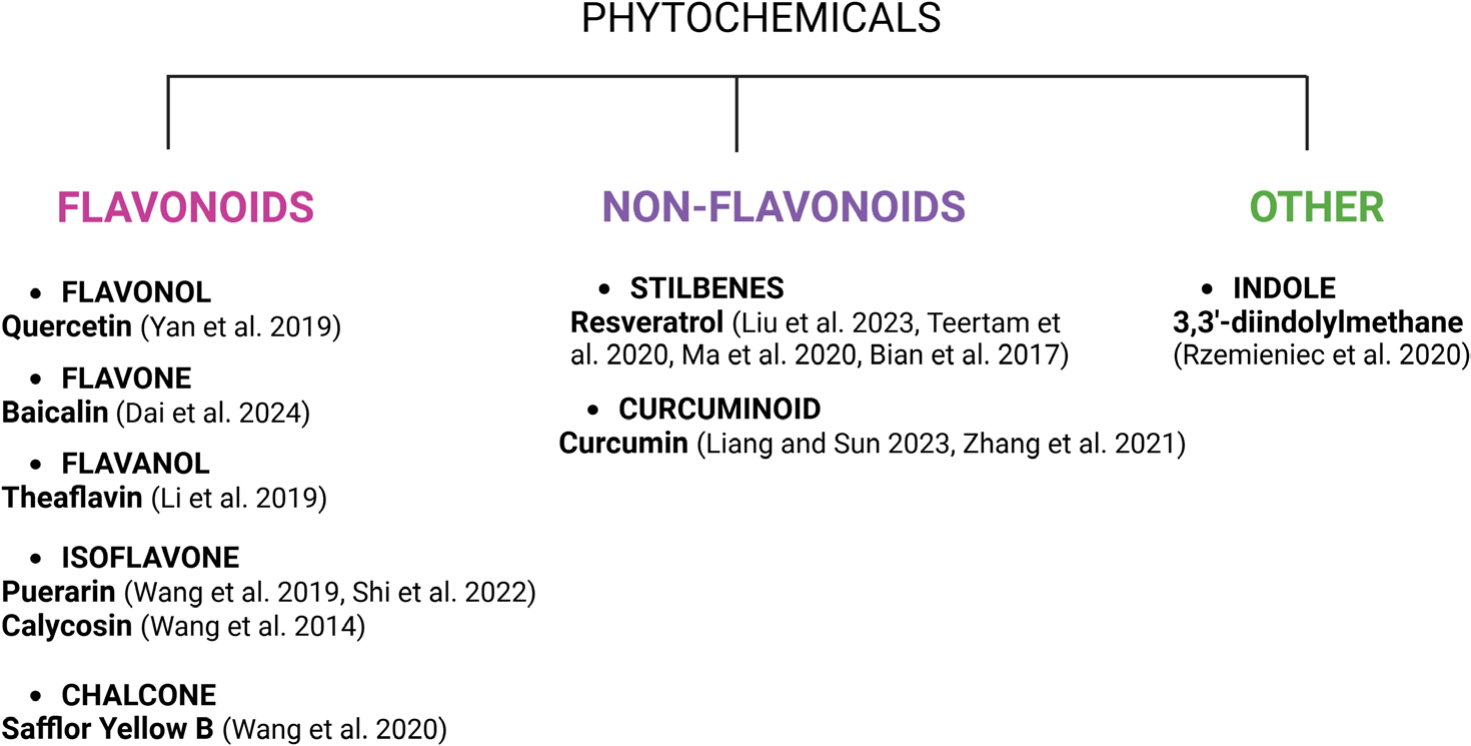
Classification of phytochemicals included in this review.

Figure 4 systematically divides the phytochemicals included in the review into different classes and subclasses. Seven publications demonstrated the beneficial effect of flavonoids in animal and cellular models of brain ischemia; in six others, it has been shown beneficial effect of non‐flavonoids, especially well‐studied were resveratrol and curcumin. One publication described the neuroprotective effect of indole, 3,3′‐diindolylmethane. Flavonoids protected the murine brain by regulation of different miRs, which in turn inhibited ischemia‐induced apoptosis, oxidative stress, and inflammation. In addition, baicalin inhibited ferroptosis, and puerarin blocked pyroptosis. Non‐flavonoids and DIM, by regulation of different miRs, inhibited ischemia‐induced apoptosis, inflammation, and oxidative stress.

#### 3.2.6 Mechanisms of Neuroprotection Induced by Phytochemicals

Table 4 summarizes the main neuroprotective pathways regulated by miRs in response to phytochemicals in animal and cellular models of brain ischemia. The most frequently described processes regulated by ischemia were apoptosis, oxidative stress, and inflammation, followed by ferroptosis, pyroptosis, and AMPK and Wnt/β‐catenin signaling pathways. Indeed, eight studies showed that in vitro and in vivo ischemia induced apoptosis, which was related to changes in miRs expression, and phytochemicals were able to inhibit this process. Apart from apoptosis, in six papers, the phytochemicals changing miRs expression inhibited ischemia‐induced oxidative stress. In three papers, anti‐inflammatory properties of plant‐derived substances were demonstrated. Besides the above‐mentioned mechanisms, single papers demonstrated inhibition of ferroptosis and pyroptosis, as well as regulation of cell survival through activation of AMPK and Wnt/β‐catenin signaling pathways.

**TABLE 4:** Overview of principal neuroprotective mechanisms of action of phytochemicals in in vitro and in vivo models of cerebral ischemia.

References	Number of papers	Mechanism involved in phytochemical‐mediated neuroprotection
(Zhang et al. 2021), (Wang, Wan, et al. 2020), (Shi et al. 2022), (Bian et al. 2017), (Teertam et al. 2020) (Rzemieniec et al. 2020), (Wang, Wan, et al. 2020), (Wang et al. 2014)	*N* = 8	Anti‐apoptotic effect
(Dai et al. 2024), (Liang and Sun 2023), (Wang, Wan, et al. 2020), (Zhang et al. 2021), (Li et al. 2019), (Liu et al. 2023)	*N* = 6	Antioxidant effect
(Ma et al. 2020), (Liang and Sun 2023), (Dai et al. 2024)	*N* = 3	Anti‐inflammation
(Dai et al. 2024)	*N* = 1	Anti‐ferroptosis

#### 3.2.7 Apoptosis

In both in vitro and in vivo studies, inhibition of apoptosis by phytochemicals was correlated with upregulation of miR‐1287‐5p (Zhang et al. 2021), miR‐214 (Wang, Ma, and Yang 2019) miR‐96 (Bian et al. 2017), miR‐223‐3p (Shi et al. 2022), miR‐149‐5p (Teertam et al. 2020), miR‐375 (Wang et al. 2014) and downregulation of miR‐181b (Rzemieniec et al. 2020) miR‐134 (Wang, Wan, et al. 2020).

#### 3.2.8 Oxidative Stress

Inhibition of ischemia‐induced oxidative stress was mediated by phytochemicals via upregulation of miR‐556‐3p (Dai et al. 2024), miR‐1287‐5p (T. Zhang et al. 2021), miR‐450b‐5p (J. H. Liu et al. 2023) and downregulation of miR‐145‐5p (Liang and Sun 2023) miR‐134 (Wang, Wan, et al. 2020), miR‐128‐3p (R. Li et al. 2019).

#### 3.2.9 Inflammation

The inflammatory processes accompanying stroke were inhibited by phytochemical‐induced increase in miR‐556‐3p (Dai et al. 2024) expression as well as decrease in miR‐145‐5p (Liang and Sun 2023) and miR‐155 (Ma et al. 2020).

#### 3.2.10 Other Mechanisms

It has been shown that Baicalin‐mediated upregulation of miR‐556‐3p evoked a decrease in the expression of ACSL4—protein closely related to ferroptosis in both in vitro and in vivo models of ischemia (Dai et al. 2024) Another flavonoid, quercetin, was able to rescue the PC‐12 cells from hypoxia‐induced damage via downregulation of miR‐122 that was accompanied by activation of prosurvival AMPK and Wnt/β‐catenin signaling pathways (Yan et al. 2019). Puerarin, by upregulation of miR‐223‐3p, not only inhibited apoptosis but also pyroptosis via decreasing levels of NLRP3 and ASC (Shi et al. 2022).

#### 3.2.11 Risk of Bias Evaluation

##### 3.2.11.1 Study In Vivo

**FIGURE 5:**
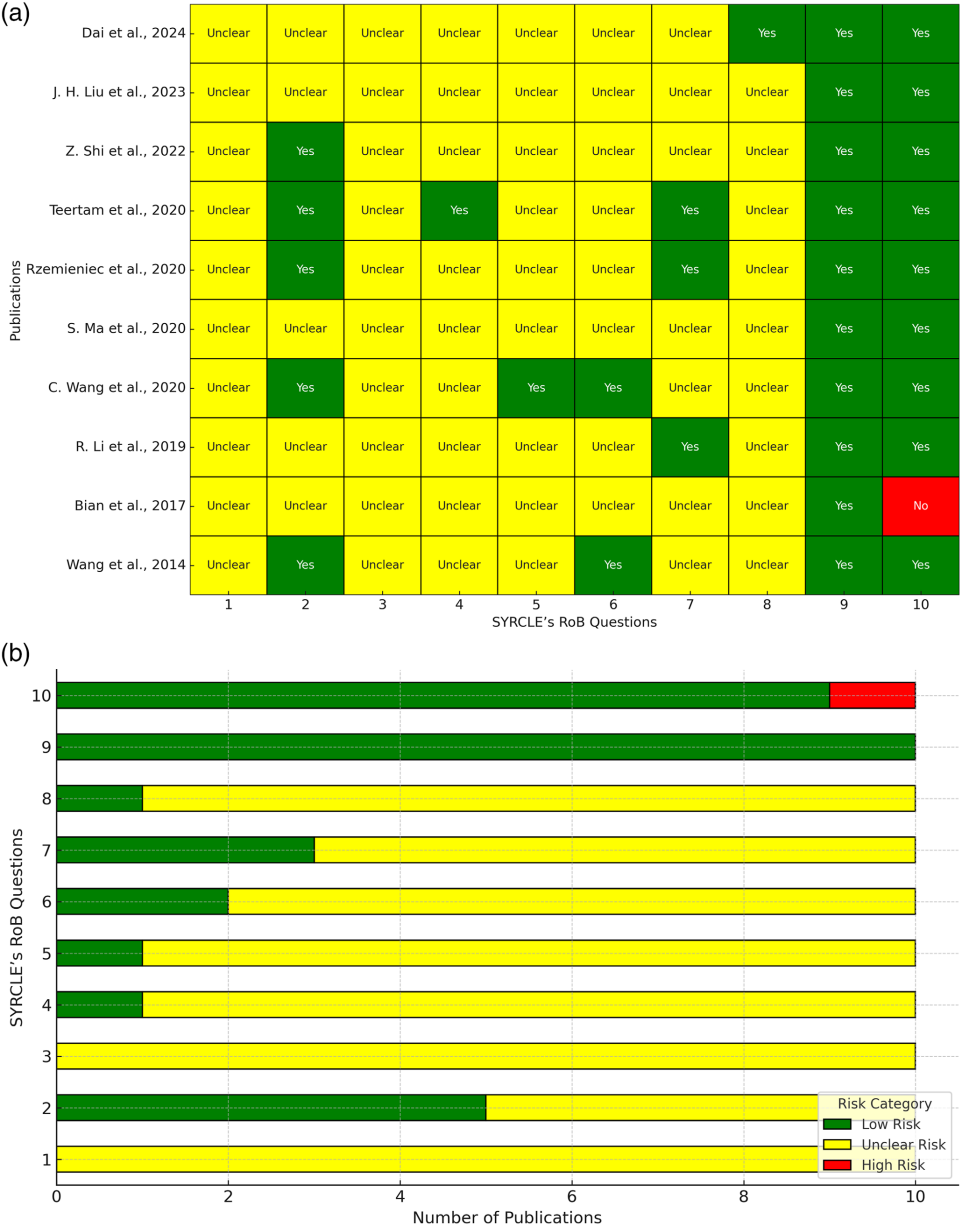
(a) Risk of bias heatmap for in vivo studies based on SYRCLE's RoB criteria. (b) Distribution of risk categories across SYRCLE's RoB domains for in vivo studies.

The SYRCLE bias assessment conducted on the 10 preclinical animal studies included in this review reveals significant gaps in reporting and methodological rigor. Across all categories, the majority of studies were rated as having an unclear risk of bias. Specifically, sequence generation (item 1) and allocation concealment (item 3) were universally unclear. Baseline characteristics in 50% of papers were rated low‐risk bias; in the other 50%, it was unclear (item 2). Performance bias and attrition bias were also poorly addressed, with 90% of studies rated as unclear risk and only 10% rated as low risk (items 4, 5, and 8). In the case of detection bias, random outcome assessment (item 6) was rated as a low risk only in 20% and blinding (item 7) in 30% of cases. Reporting bias and other risks of bias (items 9, 10) had a low risk of bias (Figure 5a,b).

##### 3.2.11.2 Study In Vitro

**FIGURE 6:**
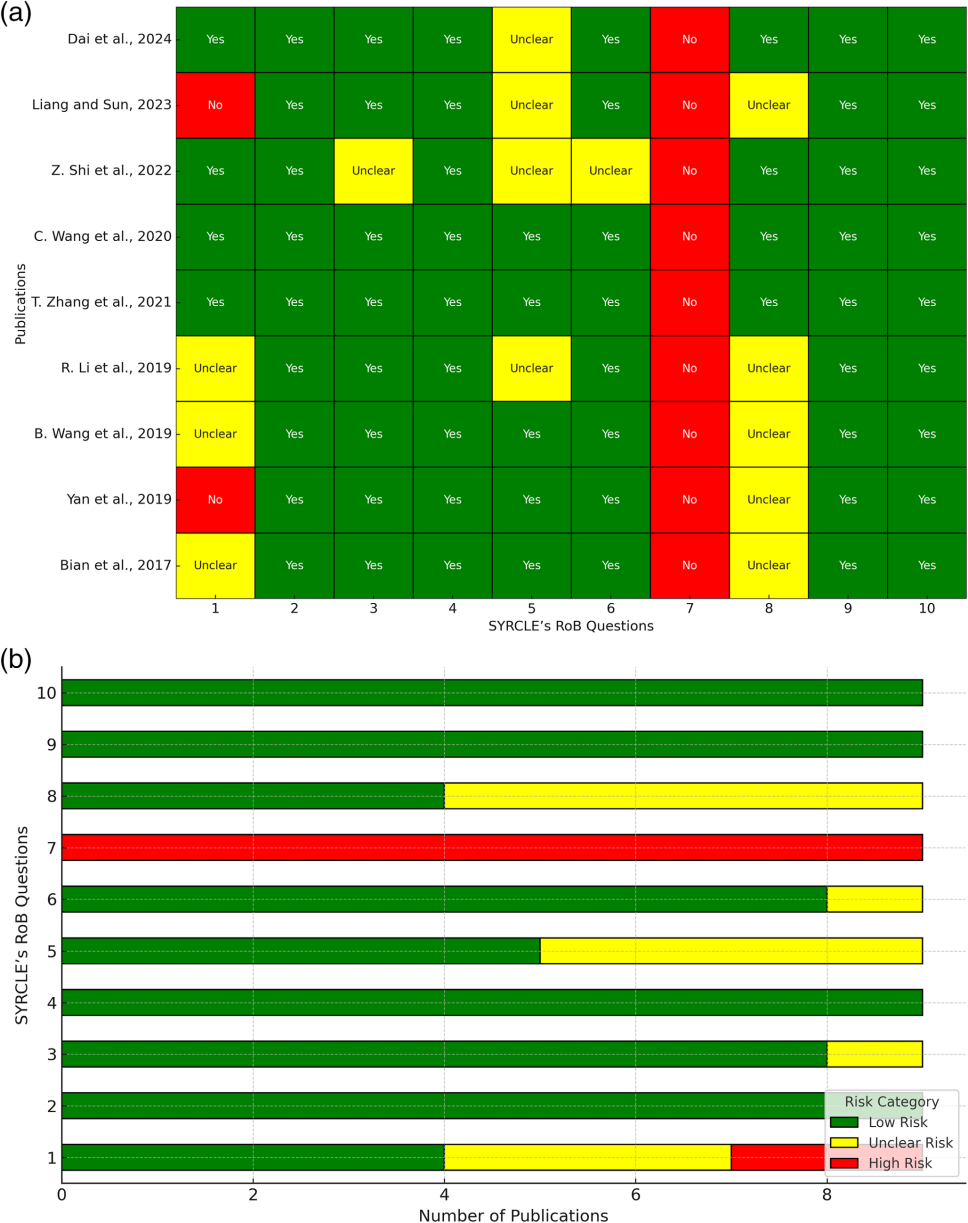
(a) Risk of bias heatmap for in vitro studies based on modified SYRCLE's RoB criteria. (b) Distribution of risk categories across modified SYRCLE's RoB domains for in vitro studies.

The modified SYRCLE bias assessment conducted on the nine preclinical in vitro studies included in this review (Figure 6a,b). After evaluation of bias for in vitro studies, it was noted that 100% of the studies showed a high risk of bias mainly due to blinding (item 7) There was also difficulty in assessing incomplete outcome data, with 54% of papers rated as unclear risk (item 8). The majority of the studies provided sufficient information and were classified as low risk of bias in relation to baseline characteristics (100% rated as a low risk, item 2), performance bias (95% rated as a low risk item 3 and 4), detection bias (method of outcome measurement‐89% rated as a low risk item 6, details of comparison group −56% rated as a low risk item 5), reporting bias, and other sources of bias (100% rated as a low risk items 9, 10).

## 4 Discussion

This review for the first time systematically examined the impact of different phytochemicals on the modulation of miRNAs and in turn their role in protecting the brain against hypoxic/ischemic injury. Both in vitro and in vivo studies indicated that phytochemicals were able to reverse the changes in expression of miRNA induced by ischemia. Phytochemicals upregulated eight different miRNAs, that is, miR‐556‐3p, miR‐223‐3p, miR‐1287‐5p, miR‐214, miR‐96, miR‐450b‐5p, miR‐149‐5p, and miR‐375, whereas downregulated seven other miRNAs, that is, miR‐145‐5p, miR‐124, miR‐128‐3p, miR‐122, miR‐181b, miR‐155, miR‐134. The changes in miRNA expression evoked by phytochemicals led to an inhibition of apoptosis, inflammation, and oxidative stress but also ferroptosis, pyroptosis, or activation of Wnt/β‐catenin and AMPK signaling pathways.

No other reviews have systematically summarized the effects of various phytochemicals on miRNA expression in the context of stroke. The already published reviews are mostly focused on the beneficial effects of polyphenols in human and/or animal models of ischemic stroke but often they exclude in vitro studies, which are valuable sources of information about the molecular mechanisms (Abdelsalam et al. 2023; Bayes et al. 2023; Pacifici et al. 2021; Zhou, Zhang, and Fan 2021). Indeed, our review summarizes both in vitro and in vivo studies and gives more detailed information about the molecular mechanisms of phytochemicals, especially in the context of miRNA. The papers collected in this review showed that the most common processes inhibited by phytochemicals through miRNA modulation are inflammation, oxidative stress, and apoptosis. This is in line with other recently published reviews, where the beneficial action of different classes of phytochemicals in experimental models of stroke was summarized. However, the authors of these papers did not characterize the involvement of miRNAs in phytochemical action (Abdelsalam et al. 2023; López‐Morales et al. 2023; Lu et al. 2024; Marques et al. 2022; Owjfard et al. 2024; Zhou, Zhang, and Fan 2021).

One of the limitations of this systematic review is a lack of studies in humans which could confirm the results obtained from the experimental models, that is, the involvement of miRNAs in the protective action of phytochemicals in stroke patients. The only review that summarizes the effect of polyphenols in post‐stroke adults demonstrated that simultaneous administration of resveratrol (Chen et al. 2016), EGCG (Wang and You 2017) or fisetin (Wang, Cao, et al. 2019) with rtPA may enlarge the therapeutic window for acute stroke patients via inhibition of inflammation. However, there is a lack of information about the expression levels of miRNA in these patients after treatment with phytochemicals (Bayes et al. 2023).

From the data reported in this Review emerges that upregulation of miR‐556‐3p by baicalin (Dai et al. 2024) or downregulation of miR‐145‐5p by curcumin (Liang and Sun 2023) and miR‐155 by resveratrol (Ma et al. 2020) is partially responsible for the attenuation of hypoxia/ischemia‐induced inflammation. A recently published Review by Lian et al. (2021) summarized the involvement of different miRNAs in neuroinflammation during the stroke; among others, they pointed to a key role of upregulation of miR‐145‐5p (Qi et al. 2017; Xie et al. 2017) and miR‐155 (Pena‐Philippides et al. 2016) in the polarization of the M1 (proinflammatory) phenotype of microglia after stroke. This is partially in line with the study of Liang and Sun (2023) and Ma et al. (2020), who showed that downregulation of these two miRNAs by phytochemicals strongly inhibited neuroinflammation via decreasing the level of proinflammatory cytokines and increasing the expression of Arginase‐1 and CD206 (Liang and Sun 2023; Ma et al. 2020). There is no other data on the regulation of miR‐556‐3p during ischemic stroke to compare with the paper of Dai et al. (2024). The only available study demonstrated that knockdown of miR‐556‐3p increased the level of cleaved caspase‐1 and IL‐1β in the parental cisplatin‐sensitive NSCLC cells (Shi et al. 2021) which is partially in line with Dai et al. 2024, who showed that hypoxia decreased the level of miR‐556‐3p and that baicalin‐induced miR‐556‐3p upregulation led to the inhibition of ferroptosis and inflammation. A different flavonoid—quercetin protected—the PC‐12 cells against hypoxia via downregulation of hypoxia‐stimulated miR‐122 and, in turn, activation of AMPK and Wnt/B‐catenin signaling pathways (Yan et al. 2019). This is partially in line with Jin et al. (2019), who confirmed that quercetin may protect the brain against ischemia through activation of Wnt signaling pathway action, but in this paper, miR‐122 was not studied (Jin et al. 2019).

Other key processes regulated by miRNAs and activated in the brain following a stroke include apoptosis and oxidative stress. The data reported in this Review indicate that the miR‐214 upregulation induced by Puerarin in rat hippocampal cells undergoing hypoxia led to inhibition of apoptosis and activation of the prosurvival Akt signaling pathway (Wang, Ma, and Yang 2019). The involvement of miR‐214 in brain protection was recently studied by other authors who showed that miR‐214‐3p promotes hypoxic‐exosome‐induced neuroprotection in mice after tMCAO by regulating the PTEN/Akt signaling pathway (Wu et al. 2024). Another phytochemical, resveratrol, exhibited anti‐apoptotic action via upregulation of miR‐96 and, in turn, downregulation of pro‐apoptotic Bax in PC‐12 cells subjected to OGD and in rat pups undergoing asphyxia (Bian et al. 2017). The protective capacity of miR‐96 was confirmed by Jin et al. 2024, who demonstrated that overexpression of miR‐96‐5p promoted less cerebral edema and a smaller infarct area in mice after MCAO (Jin et al. 2024). However, in this paper, the authors did not study either the effect of raloxifene or apoptosis. Treatment of rats with resveratrol just before tMCAO led to upregulation of miR‐149‐5p and inhibition of apoptosis (Teertam et al. 2020). Similarly, overexpression of miR‐149‐5p reduced MCAO/R‐induced infarct volume, neurological score, and brain water content in mice and OGD/R‐induced cortical neuron apoptosis and inflammation (Wang, Xu, and Wang 2022). Treatment with phytoestrogens calycosin (Wang et al. 2014) or puerarin (Shi et al. 2022; Wang, Ma, and Yang 2019) resulted in great neuroprotection against hypoxia/ischemia damage correlated with miR‐375 or miR‐214 and miR‐223‐3p upregulation and inhibition of apoptosis. A similar result was obtained by Beyer et al. 2017, who showed that treatment of rats with estrogen or progesterone after tMCAO strongly upregulated miR‐375 expression in the rat brain; however, it downregulated miR‐214 and miR‐223 (Beyer et al. 2017). This discrepancy could be related to different substances and ischemia models used as well as the different timepoints of the measurement of miRs.

In this review, downregulation of miR‐134 by Safflor Yellow B protected rat neurons against hypoxia/ischemia via activation of CREB level (Wang, Wan, et al. 2020) and, in turn, inhibition of apoptosis and oxidative stress. The same mechanism of protection was observed by Yang et al. (2020), who demonstrated that inhibition of miR‐134 expression reduces infarct‐induced apoptosis via Creb1 upregulation (Yang et al. 2020). The involvement of the above‐mentioned miRNAs in stroke pathology was confirmed by Salman et al. (2022) who showed strong downregulation of miR‐375 and upregulation of miR‐134 in the serum of patients suffering from acute ischemic stroke, which points to the important role of these miRs in stroke diagnosis and treatment. Apart from downregulation of miR‐134, downregulation of miR‐181b‐5p also seems to be crucial for brain protection from ischemia‐induced apoptosis. Indeed, DIM decreased expression of miR‐181b‐5p and protected rat pups' brains against perinatal asphyxia via inhibition of apoptosis (Rzemieniec et al. 2020). Other authors showed that miR‐181b‐5p downregulation by isosteviol sodium led to inhibition of apoptosis both in vitro and in vivo in a model of cerebral ischemia (Zhang et al. 2018). Apart from the involvement of curcumin in inhibition of neuroinflammation and oxidative stress through miR‐145‐5p downregulation after ischemia (Liang and Sun 2023), it has been shown that curcumin‐induced upregulation of miR‐1287‐5p inhibits apoptosis and ROS formation in SH‐SY5Y cells subjected to OGD (Zhang et al. 2021). There are no other studies to compare these results with. Oxidative stress was also inhibited by resveratrol that upregulated expression of miR‐450b‐5p and, in turn, inhibited KEAP1 and stimulated Nrf2 (Liu et al. 2023). This mechanism of action of resveratrol was also confirmed in microglial cells treated with rotenone or in endothelial cells subjected to hypoxia/ischemia (Song et al. 2021; Sun et al. 2021). The elevated level of miR‐128‐3p was detected in circulating lymphocytes, neutrophils, and plasma of patients with AIS compared with healthy individuals (Liu et al. 2019). Indeed, downregulation of miR‐128‐3p by theaflavin led to a reduction of oxidative stress by upregulation of the Nrf2 signaling pathway both in an in vitro and in vivo model of cerebral ischemia (Li et al. 2019). There are no other papers that observed the same effect of theaflavin in animal or cellular models of brain ischemia. The only study comes from the model of renal–ischemia reperfusion (I/R) injury model, where theaflavin exerted a protective effect via activation of the Nrf2‐NQO1/HO‐1 pathway (Li et al. 2021).

This review for the first time in a systematic way summarizes the protective mechanisms of action of phytochemicals with special focus on miRNAs in cellular and animal models of cerebral ischemia. However, there are several limitations in the published literature which must be underlined. First, most of the cited studies in vivo were performed in tMCAO that do not perfectly mimic the stroke in humans. Indeed, in 95% of patients suffering from Large Vessel Occlusion strokes, the occlusion is permanent (McBride and Zhang 2017). Using the tMCAO for testing drugs may give rise to positive results that are not replicable in humans. Secondly, in a large number of papers, the authors pre‐treated the cells with phytochemicals and then performed OGD, which do not have great clinical implications; thus, most of the patients reach the hospital with ongoing stroke. Thirdly, even if some publications included in this review tested the same compound, for example, resveratrol, curcumin, or puerarin, the different miRNAs were assessed in these papers, which may weaken the final conclusion. Finally, in this review, we did not include the papers that describe the involvement of long non‐coding RNAs (lncRNAs) and circular RNAs (circRNAs) which could give us a broader view on the involvement of all non‐coding RNAs in the mechanisms of action of phytochemicals during the stroke.

## 5 Conclusions

Our analysis indicates that phytochemicals, through the modulation of miRNA expression, effectively inhibit H/I‐induced apoptosis, neuroinflammation, and oxidative stress, highlighting the complexity of their protective effects. By targeting multiple pathways involved in stroke pathology, phytochemicals present a multifaceted approach for treating cerebral ischemia. This review provides a comprehensive overview of current research on phytochemicals‐mediated miRNA modulation, examining both the underlying molecular mechanisms and the potential future clinical applications.

## 6 Future Perspectives

The modulation of miRNAs by phytochemicals represents a promising strategy for developing new treatments for cerebral ischemia. Future perspectives on the development of phytochemical‐based therapies highlight the potential of these compounds to modulate specific miRNAs associated with critical pathological processes such as apoptosis, inflammation, oxidative stress, and ferroptosis. The regulatory effects of phytochemicals, including curcumin's influence on miR‐1287‐5p and resveratrol's modulation of miR‐155, suggest their capacity to target RNA pathways effectively, presenting a promising approach for therapeutic intervention in ischemic conditions. To realize this potential, advanced delivery systems such as nanoparticles or exosome‐based carriers could be employed to enhance the stability, bioavailability, and targeted delivery of these compounds to ischemic brain regions. Combining phytochemical‐induced miRNA modulation with synthetic miRNA mimics or inhibitors offers an opportunity to amplify therapeutic efficacy, especially by enhancing neuroprotective effects and reducing inflammation. Additionally, miRNAs modulated by phytochemicals could serve as biomarkers, providing diagnostic and prognostic insights and enabling personalized therapeutic strategies. Given that the included studies are based on cellular and animal models of brain ischemia, further clinical trials in human stroke patients are needed to validate these findings. This review serves as a valuable starting point for identifying the most promising phytochemicals to be advanced into clinical studies. Due to their natural properties, phytochemicals hold the potential to enhance outcomes for patients suffering from stroke and other ischemic conditions.

## Author Contributions

**Joanna Rzemieniec:** conceptualization, data curation, formal analysis, funding acquisition, project administration, supervision, writing – original draft, writing – review and editing. **Mirko Marino:** conceptualization, data curation, methodology, supervision, writing – original draft, writing – review and editing. **Benedetta Mercuriali:** data curation, investigation, methodology, writing – review and editing. **Laura Castiglioni:** writing – review and editing. **Paolo Gelosa:** writing – review and editing. **Majeda Muluhie:** writing – review and editing. **Cristian Del Bo':** methodology, writing – review and editing. **Patrizia Riso:** validation, writing – review and editing. **Luigi Sironi:** supervision, validation, writing – review and editing.

## Conflicts of Interest

The authors declare no conflicts of interest.

## Supporting information


**TABLE S1:** Modified SYRCLE's RoB tool.

## Data Availability

Data openly available in a public repository that issues datasets with DOIs.
