# Supplementary material for: Modulation of miRNAs by Phytochemicals in Cerebral Ischemia: A Systematic Review of In Vitro and In Vivo Studies
Source: Phytother Res. 2025 Aug 13;39(9):4327–47. doi: 10.1002/ptr.70062 (PMC12423494; doi:10.1002/ptr.70062)
Supplement: Supplementary file 1 — TABLE S1: Modified SYRCLE's RoB tool. [file PTR-39-4327-s001.docx]

**Modified SYRCLE’s RoB tool.**

The SYRCLE’s RoB tool was adjusted for application to *in vitro* research. The resulting RoB tool for *in vitro* studies included in our review contains 10 entries, as shown in Table S1.

Table S1. Modified SYRCLE’s RoB tool

| Item | Type of bias | Domain | Description of domain | Review authors judgment |
| --- | --- | --- | --- | --- |
| 1 | Selection bias | Sample size calculation | Details of the sample size calculation method (software, formula and parameters used to calculate the sample size) should be clearly indicated. | Was the sample size properly calculated and  applied? |
| 2 | Selection bias | Baseline characteristics | Details of cell lines used | Is it clear whether the cell lines used are primary  cell cultures or continuous cell lines |
| 3 | Performance  bias | Detailed explanation of  intervention and the treatment | The intervention procedure must indicate the time of hypoxia/ischemia and reoxygenation. The treatment must indicate the concentration, the vehicle and the incubation time of the substances administered | Were interventions administered to cells in a consistent and standardized manner throughout  the experiment? |
| 4 | Performance  bias | Detailed explanation of  culture conditions | Provide any information relating to culture conditions | Are culture conditions comparable between  different studies or groups within studies? |
| 5 | Detection bias | Details of comparison  group | Details of comparison groups (positive, negative control) should be clearly specified. | Do appropriate controls are included? |
| 6 | Detection bias | Method of measurement  of outcome | The procedure and rationale for the choice of method, and how the results were evaluated should be clearly stated | Were the statistical analysis clearly specified? |
| 7 | Detection bias | Blinding | Details on all measures used, if any, to prevent outcome  assessors from knowing which intervention each cell culture received. Provide any information regarding the effectiveness of the planned blinding | Was the outcome assessor blinded? (*) |
| 8 | Attrition bias | Incomplete outcome data | Describe the completeness of outcome data for each main  outcome, including attrition and exclusions from the analysis | Were incomplete outcome data adequately addressed? (*) |
| 9 | Reporting bias | Selective outcome  reporting | State how selective outcome reporting was examined and what was found | Are reports of the study free of selective outcome reporting? (*) |
| 10 | Other | Other sources of bias | State any important concerns about bias not covered by other domains in the tool | Was the study apparently free of other problems that could result in a high risk of bias? (*) |
